# Regulation of Yeast-to-Hyphae Transition in Yarrowia lipolytica

**DOI:** 10.1128/mSphere.00541-18

**Published:** 2018-12-05

**Authors:** Kyle R. Pomraning, Erin L. Bredeweg, Eduard J. Kerkhoven, Kerrie Barry, Sajeet Haridas, Hope Hundley, Kurt LaButti, Anna Lipzen, Mi Yan, Jon K. Magnuson, Blake A. Simmons, Igor V. Grigoriev, Jens Nielsen, Scott E. Baker

**Affiliations:** a Chemical & Biological Process Development Group, Pacific Northwest National Laboratory, Richland, Washington, USA; b Environmental Molecular Sciences Division, Pacific Northwest National Laboratory, Richland, Washington, USA; c Department of Biology and Biological Engineering, Chalmers University of Technology, Göteborg, Sweden; d DOE Joint Genome Institute, Walnut Creek, California, USA; e Joint BioEnergy Institute, Lawrence Berkeley National Laboratory, Berkeley, California, USA; f Novo Nordisk Foundation Center for Biosustainability, Technical University of Denmark, Lyngby, Denmark; Carnegie Mellon University

**Keywords:** *Yarrowia*, dimorphic, genomics, molecular genetics, morphology, signaling

## Abstract

The yeast Yarrowia lipolytica undergoes a morphological transition from yeast-to-hyphal growth in response to environmental conditions. A forward genetic screen was used to identify mutants that reliably remain in the yeast phase, which were then assessed by whole-genome sequencing. All the *smooth* mutants identified, so named because of their colony morphology, exhibit independent loss of DNA at a repetitive locus made up of interspersed ribosomal DNA and short 10- to 40-mer telomere-like repeats. The loss of repetitive DNA is associated with downregulation of genes with stress response elements (5′-CCCCT-3′) and upregulation of genes with cell cycle box (5′-ACGCG-3′) motifs in their promoter region. The stress response element is bound by the transcription factor Msn2p in Saccharomyces cerevisiae. We confirmed that the Y. lipolytica *msn2* (Yl*msn2*) ortholog is required for hyphal growth and found that overexpression of Yl*msn2* enables hyphal growth in *smooth* strains. The cell cycle box is bound by the Mbp1p/Swi6p complex in S. cerevisiae to regulate G_1_-to-S phase progression. We found that overexpression of either the Yl*mbp1* or Yl*swi6* homologs decreased hyphal growth and that deletion of either Yl*mbp1* or Yl*swi6* promotes hyphal growth in *smooth* strains. A second forward genetic screen for reversion to hyphal growth was performed with the *smooth-33* mutant to identify additional genetic factors regulating hyphal growth in Y. lipolytica. Thirteen of the mutants sequenced from this screen had coding mutations in five kinases, including the histidine kinases Yl*chk1* and Yl*nik1* and kinases of the high-osmolarity glycerol response (HOG) mitogen-activated protein (MAP) kinase cascade Yl*ssk2*, Yl*pbs2*, and Yl*hog1*. Together, these results demonstrate that Y. lipolytica transitions to hyphal growth in response to stress through multiple signaling pathways.

**IMPORTANCE** Many yeasts undergo a morphological transition from yeast-to-hyphal growth in response to environmental conditions. We used forward and reverse genetic techniques to identify genes regulating this transition in Yarrowia lipolytica. We confirmed that the transcription factor Yl*msn2* is required for the transition to hyphal growth and found that signaling by the histidine kinases Yl*chk1* and Yl*nik1* as well as the MAP kinases of the HOG pathway (Yl*ssk2*, Yl*pbs2*, and Yl*hog1*) regulates the transition to hyphal growth. These results suggest that Y. lipolytica transitions to hyphal growth in response to stress through multiple kinase pathways. Intriguingly, we found that a repetitive portion of the genome containing telomere-like and rDNA repeats may be involved in the transition to hyphal growth, suggesting a link between this region and the general stress response.

## Abstract (precis)

Many yeasts undergo a morphological transition from yeast-to-hyphal growth in response to environmental conditions. We used forward and reverse genetic techniques to identify genes regulating this transition in Yarrowia lipolytica. We confirmed that the transcription factor Yl*msn2* is required for the transition to hyphal growth and found that signaling by the histidine kinases Yl*chk1* and Yl*nik1* as well as the MAP kinases of the HOG pathway (Yl*ssk2*, Yl*pbs2*, and Yl*hog1*) regulates the transition to hyphal growth. These results suggest that Y. lipolytica transitions to hyphal growth in response to stress through multiple kinase pathways. Intriguingly, we found that a repetitive portion of the genome containing telomere-like and rDNA repeats may be involved in the transition to hyphal growth, suggesting a link between this region and the general stress response.

## INTRODUCTION

Many fungi harbor the ability to grow in either a yeast, pseudohyphal, or hyphal form (1). Morphological plasticity allows fungi to adapt to and invade new environments in response to external conditions. This trait, while essential for fungi in natural environments, can be problematic for their use in industrial settings, such as cultivation in bioreactors. The morphological switch between yeast and hyphal growth can be initiated by nutritional, pH, temperature, and osmolarity cues (2–5). Industrial utilization of dimorphic yeasts presents a particular challenge, as maximum economic efficiency demands that bioreactors be run at high temperature and osmolarity using low-quality nutrients, all of which may initiate the switch to hyphal growth.

Dimorphism is common in many species of ascomycete yeasts and has been most thoroughly studied in the genetic model Saccharomyces cerevisiae and the closely related opportunistic pathogen Candida albicans where the switch to hyphal growth is important for infection (6). Environmental signals controlling hyphal growth regulate specific genetic outputs through kinase cascades and calcium signaling pathways. The adenylate cyclase Cyr1p is required for hyphal growth in yeasts (7, 8) and signals through protein kinase A (PKA) to the transcription factor Efg1p to promote the yeast-to-hyphae transition (9, 10). Two mitogen-activated protein kinase (MAPK) cascades integrate signals from different sources to position and regulate filamentous growth in yeasts. The kinase Ste20p responds to the GTPase Cdc42p and activates the Ste11p/Ste7p/Kss1p MAPK cascade to control polarized growth and bud site selection (5, 11, 12), while the Ssk2p/Pbs2p/Hog1p MAPK cascade responds to osmotic and oxidative stress in S. cerevisiae and C. albicans and regulates the yeast-to-hyphae transition in both species (10, 13, 14).

Yarrowia lipolytica is a model industrial ascomycete yeast distantly related to S. cerevisiae and C. albicans (15). The yeast-to-hyphae transition in this species has been examined by proteomics and transcriptomics (16, 17) and has given clues to the proteins involved. The transition is regulated by a number of transcription factors, including those encoded by *znc1* (18), *tec1* (19), *hoy1* (20), and the histone deacetylase complex component gene *sin3* (21). The Y. lipolytica *msn2* (Yl*msn2*) homolog (originally identified as *mhy1* in Y. lipolytica) is critical for the yeast-to-hyphae transition and is positively regulated by the kinase Rim15p which itself is repressed by the Tor nitrogen signaling pathway (22, 23). As in other yeasts, Ras GTPases (Ras1p and Ras2p) are essential for the dimorphic transition and also likely signal through the transcription factor Msn2p (24, 25).

In this study, we isolated strains of Y. lipolytica that fail to undergo the yeast-to-hyphae transition. These *smooth* colony mutants do not form hyphae in a bioreactor, making them more amenable as industrial bioproduction hosts. We characterized the mutations present in the mutants obtained and mutations that promote the transition to hyphal growth in a *smooth* strain to further elucidate the signaling pathways regulating dimorphic growth in Y. lipolytica.

## RESULTS

### Isolation of Y. lipolytica mutants lacking filamentous growth.

**FIG 1:**
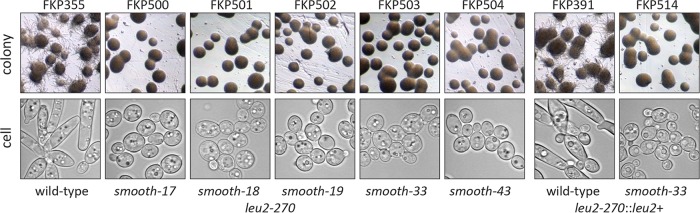
Isolation of Y. lipolytica mutants that lack filamentous growth. Approximately 500,000 colonies were screened for *smooth* morphology with no visible hyphae. From strain FKP355, five mutant strains were isolated that exhibit growth only as yeast (FKP500 to FKP504). The *leu2-270* mutation was complemented in strain FKP503 to construct FKP514 and confirm the phenotype in a prototrophic strain. Confocal microscopy confirmed yeast phase growth and lack of elongated cells or pseudohyphae in auxotrophic and prototrophic *smooth* strains.

Y. lipolytica strain FKP355 was passaged to allow accumulation of mutations and screened for lack of filamentous growth from large colonies. Small slow growing colonies often did not produce hyphae or did so only under certain conditions or after an extended period of time. Approximately 500,000 colonies were screened from which 65 mutants were isolated that did not appear to make hyphae. After isolation, these mutants were further tested for filamentous growth after 2 weeks of incubation on YNB, YNB150, and YPD agar (see Materials and Methods), as well as YPD and YNB150 liquid medium for microscopic analysis. From those mutants, five *smooth* mutants (*smooth-17*, *smooth-18*, *smooth-19*, *smooth-33*, and *smooth-43*) were identified that did not undergo transition to hyphal growth morphology under any of the conditions tested (Fig. 1). Many of the original 65 isolates produced short invasive hyphae into the agar or grew slowly and were not considered further in this work. Approximately 100,000 colonies from each of the five mutants were screened for reversion to hyphal growth habit from the *smooth* phenotype. No revertants (<0.0002%) from any of the mutants were identified, confirming genetic stability.

### Identification of mutations in Y. lipolytica mutants lacking filamentous growth.

**FIG 2:**
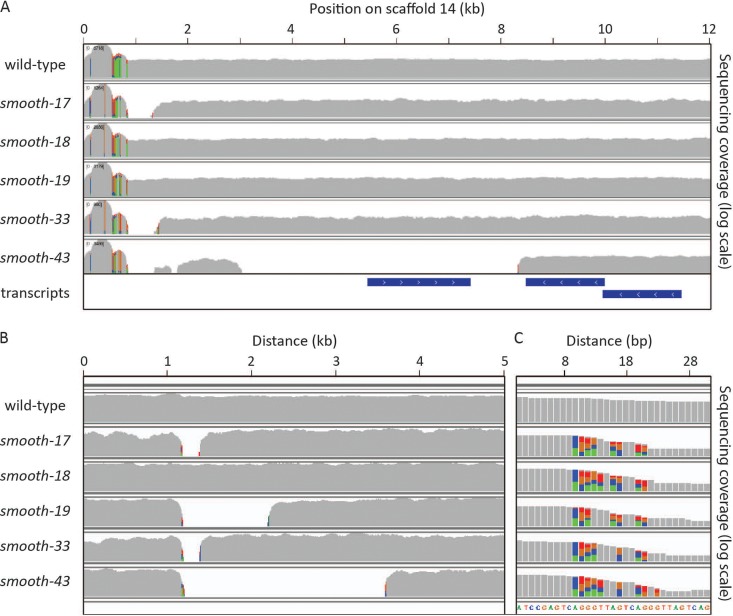
*smooth* strains have mutations in repetitive regions of the genome. Coverage from high-throughput 150-bp paired-end Illumina sequencing from strain FKP355 (wild type) and five *smooth* mutant strains. Colored bases indicate polymorphic loci where reads align with SNPs at a rate greater than that expected from incorrect base calls. (A) Regions with no coverage are detected in *smooth-17*, *smooth-33*, and *smooth-43* mutants at the end of scaffold 14 after alignment to the FKP355 reference genome. (B) Raw PacBio reads with homology to the single-copy region at the end of scaffold 14 (from 1 to 12 kb) were reassembled and analyzed for mutations not detected from the curated genome assembly. An example of an alternative assembly of the region detects a deletion in *smooth-19* not seen in the reference assembly. (C) All five *smooth* mutants exhibit a different polymorphism rate than the wild-type rate at a transition point between a high-copy-number transposon-containing region and a moderate-copy-number region of short, tandem repeats.

Each of the five mutants lacking filamentous growth and the wild-type parent (FKP355) were sequenced using Illumina paired-end 150-base-pair sequencing to an average depth of >13× to identify the causative mutations. This initial search revealed few mutations limited to a single nucleotide polymorphism (SNP) affecting a tRNA in *smooth-17*, a deletion in gene Yali0F20592g in *smooth-19*, and a noncoding SNP in *smooth-43*. None of these candidate genes complemented the *smooth* phenotype when expressed from an autonomously replicating plasmid (data not shown). To better assess the mutants, genomic DNA from strain FKP355 was sequenced on the PacBio platform to a depth of 279×, assembly and annotation of which are available at http://genome.jgi.doe.gov/Yarlip1/Yarlip1.info.html. Using this assembled genome allowed us to search for gaps in read coverage in the mutants and resulted in identification of deletions in *smooth-17*, *smooth-33*, and *smooth-43* strains. Interestingly, the deletions are in the same general location near the end of scaffold 14 in all three of these *smooth* mutants (Fig. 2A). Analysis of the mutated region of scaffold 14 revealed that it ends in an array of polymorphic 5′-TTAGTCAGGG-3′ tandem DNA repeats previously described as the telomere repeat sequence in Y. lipolytica (26). Exceptionally high sequencing read depth at this locus suggests that it is highly repetitive and underrepresented in the genome assembly. We therefore sought to explore the possibility of alternative assemblies of the DNA at the end of scaffold 14 to better understand the composition of this mutated locus.

We hypothesized that the length of the repetitive DNA present at the end of scaffold 14 is much longer than the ∼800 bp represented in the genome assembly and used long PacBio sequencing reads from strain FKP355 to test this. We identified 3,786 reads ranging from 117 to 29,910 bp in length (average of 3,548 and median of 2,331 bp) that aligned to a unique portion of the genome near the end of scaffold 14. This subset of reads was assembled using Canu (27) to assess the minimum length and content of the repetitive DNA adjacent to the end of the unique part of scaffold 14 without assembly interference from additional repetitive reads from different loci. From these reads, seven alternative contigs to scaffold 14 were assembled that mapped to a variety of scaffolds within the reference genome, confirming the repetitive nature of the locus. We aligned the 150-bp Illumina sequencing reads from strain FKP355 and the five *smooth* mutants to these new contigs and identified mutations. Interestingly, six out of seven of these alternative contigs harbor mutations in at least one of the *smooth* strains (Fig. 2B and C), while the seventh is a complete assembly of the ribosomal DNA (rDNA) locus (18S, 5.8S, and 28S rRNA) (28) with adjacent 5′-TTAGTCAGGG-3′ tandem repeats. These results suggest that all five *smooth* mutants harbor mutations in a related locus with short tandem repeats and rDNA repeats.

### Repeat analysis of the FKP355 genome and *smooth* mutants.

Given that the mutations identified in the *smooth* strains affected tandem repetitive DNA, we decided to more thoroughly assess the repetitive DNA content of the FKP355 genome. To avoid biases from the genome assembly process, we again examined the DNA directly in 150-bp Illumina sequencing reads from strain FKP355 for the presence of tandem repeats to identify and define all the telomere-like repetitive sequences present in this strain. All possible tandem duplications of unit size 1 to 75 bp were quantified in the raw sequencing reads, and the copy number of each repeat was estimated as follows:

$$\text{Copy}\;\text{number}=\frac{\text{Times}\;\text{found}\times \text{Genome}\;\text{size}}{\text{Total}\;\text{reads}\times (\text{Read}\;\text{length}-\text{Repeat}\;\text{length}+1)}$$

**FIG 3:**
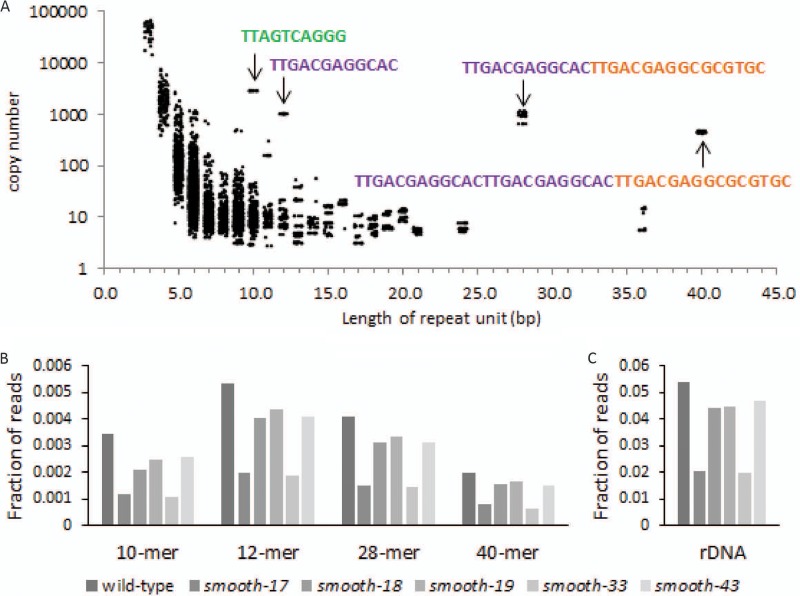
*smooth* strains have reduced repetitive DNA content. (A) Illumina 150-bp sequencing reads from strain FKP355 were systematically analyzed for the presence of all possible tandem duplications with a repeat unit length of 1 to 75 bp and quantified. Identification of phased repeat units with similar coverage was used to infer arrays of tandem repeats longer than a simple duplication. Colors indicate overlapping sequence motifs found in similar repeat sequences. (B) The fraction of 150-bp sequencing reads from the wild-type and *smooth* strains containing high-frequency tandem duplications of 10, 12, 28, and 40 bp in length. (C) The fraction of 150-bp sequencing reads from the wild-type and *smooth* strains that align to the FKP355 rDNA repeat.

The most overrepresented tandem repeat sequences identified in the FKP355 genome correspond to the 5′-TTAGTCAGGG-3′ 10-mer found at the end of scaffold 14 as well as derivations on a 5′-TTGACGAGGCAC-3′ 12-mer on its own and in combination with a 5′-TTGACGAGGCGCGTGC-3′ 16-mer (Fig. 3A). A number of low-copy-number polymorphic variations on these repeat sequences were also identified. Long PacBio sequencing reads from strain FKP355 containing tandem duplications of the 5′-TTAGTCAGGG-3′ repeat unit were identified and aligned to the FKP355 genome assembly to identify additional repetitive and/or single-copy loci adjacent to this repeat array but found the end of scaffold 14 as the only nonrepetitive assembled portion of the genome adjacent to a 5′-TTAGTCAGGG-3′ repeat array. This result suggests that either a single large 5′-TTAGTCAGGG-3′ repeat array is present in the genome or that additional 5′-TTAGTCAGGG-3′ repeat arrays are present but bordered by alternative unassembled repetitive DNA sequences consistent with subtelomere structural arrangements in yeast (29) and humans (30).

We identified individual long PacBio reads from strain FKP355 containing each of the short tandem 10- to 40-bp repeat elements as well as those mapping to the assembled rDNA locus to test how often different repeat sequences cooccurred. Roughly 11% of the long reads with a tandem 10-mer 5′-TTAGTCAGGG-3′ repeat also mapped to the rDNA locus, while a higher percentage of the reads with a 5′-TTGACGAGGCAC-3′ derived tandem repeat (49% of the 12-mer, 52% of the 28-mer, and 32% of the 40-mer) also mapped to the rDNA locus. Together, these results suggest that short tandem repetitive DNA is interspersed with the rDNA repeats.

We hypothesized that changes in the repetitive DNA content of the genome might underlie the *smooth* phenotype. Thus, the number of Illumina sequencing reads containing each of the different repeat units was assessed in the wild-type strain and each of the *smooth* mutants to quantify repetitive DNA content in a reference genome agnostic manner (Fig. 3B). All the *smooth* mutants have a decrease in short tandem repetitive DNA content with the greatest losses in the *smooth-17* and *smooth-33* mutants. These two mutants present a similar deletion when mapped to the FKP355 reference genome (Fig. 2). The number of reads mapping to the rDNA locus was also assessed, as there appears to be at least some rDNA that is genetically linked to the end of scaffold 14 as well as the 10-mer 5′-TTAGTCAGGG-3′ tandem repeats. All the *smooth* mutants have relatively fewer reads that map to the rDNA locus in a ratio similar to that of the short tandem repeat sequences (Fig. 3C). This suggests that the rDNA and the short tandem repeats together make up a repetitive part of the genome that is lost in the *smooth* mutants. We unsuccessfully attempted to reconstruct these complex mutations by transforming the wild-type parent (FKP355) with resistance marker constructs designed to randomly replace large tracts of repetitive DNA (data not shown). Thus, while the loss of repetitive DNA in the *smooth* mutants is intriguing, it has not been verified to be the cause of the *smooth* phenotype.

### Transcriptome analysis of a *smooth* mutant.

**FIG 4:**
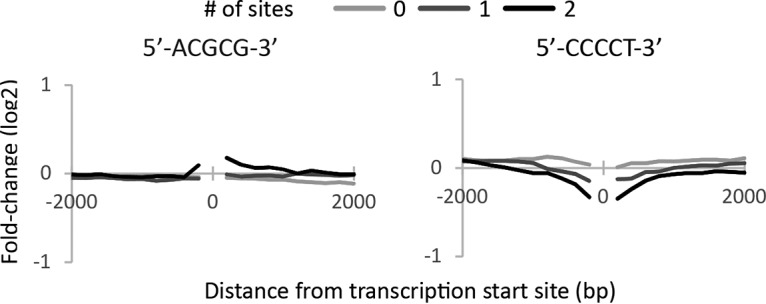
Effect of *smooth-33* on expression of genes with specific DNA motifs near their transcription start site. The number of ACGCG and CCCCT motifs on each strand of DNA was determined (from 0 to 2 sites) between the transcription start site (labeled 0) and a given distance. The given distances shown are 200 to 2,000 bp in 200-bp intervals, both up- and downstream of the transcription start site. For each interval, the average difference in expression between FKP514 (*smooth-33*) and FKP391 (wild type) during chemostat cultivation is shown. Note that the presence of more CCCCT motifs close to the transcription start site is generally associated with decreased expression in the *smooth-33* mutant, while the presence of more than one ACGCG site very near and 3′ of the transcription start site is associated with increased expression in the *smooth-33* mutant.

We compared gene expression from a prototrophic *smooth-33* mutant (FKP514) to a prototrophic wild-type strain of the same genetic background (FKP391) in chemostat culture to assess the effect on gene expression. Differentially expressed genes were analyzed for enrichment of Gene Ontology terms to assess specific biological processes perturbed in the *smooth-33* mutant (Table 1). Genes associated with DNA replication and repair as well as transcriptional regulation are more highly expressed in the *smooth-33* strain, while genes associated more generally with signaling, as well as membrane and cell wall biochemistry are downregulated. The promoter regions of differentially expressed genes were analyzed for enrichment of short DNA motifs to identify regulatory pathways acting through sequence-specific DNA-protein interactions. Genes upregulated in the *smooth-33* mutant are enriched for 5′-ACGCG-3′ motifs in their promoters, while genes downregulated in the *smooth-33* mutant are enriched for 5′-CCCCT-3′ motifs in their promoter region (E value < 0.05). We assessed the differential expression levels of genes with zero or more of these motifs near the transcription start site to confirm a specific effect on gene expression (Fig. 4). The presence of 5′-ACGCG-3′ near the transcription start site has a slight positive effect on expression level in the *smooth-33* mutant. This is primarily associated with the presence of no less than two 5′-ACGCG-3′ sites within 200 bp 5′ and 1,000 bp 3′ of the transcription start site. The presence of 5′-CCCCT-3′ both 5′ and 3′ of the transcription start site is associated with a large negative effect on the expression level in the *smooth-33* mutant in a manner that increases with the number of 5′-CCCCT-3′ sites.

**TABLE 1:** Enriched Gene Ontology terms in the *smooth-33* mutant^*a*^

GO term	FDR
Upregulated in the *smooth-33* mutant	
DNA repair	1.2E−05
Regulation of transcription from RNA polymerase II promoter	5.7E−04
DNA recombination	5.8E−03
DNA replication initiation	1.7E−02
Cell cycle process	3.3E−02
Mismatched DNA binding	3.3E−02
Nucleosome assembly	4.2E−02

Downregulated in the *smooth-33* mutant	
Small-GTPase-mediated signal transduction	1.8E−03
Steroid biosynthetic process	4.0E−03
GTP catabolic process	4.9E−03
Cytokinesis	1.7E−02
Nucleocytoplasmic transport	2.7E−02
Cellular lipid metabolic process	3.5E−02
Oxygen transport	3.6E−02
Membrane raft organization	3.6E−02
Chitin metabolic process	4.2E−02
Response to toxic substance	4.2E−02
Regulation of molecular function	4.5E−02
Fungal-type cell wall organization	4.5E−02
Microtubule-based movement	4.9E−02

a Analysis of the top 1,000 up- and downregulated genes identified biological process Gene Ontology (GO) terms specifically overrepresented in the *smooth-33* mutant (false-discovery rate [FDR] of <0.01).

We searched the Jaspar core fungal motifs database (31) for proteins that are known to interact with either of these motifs. A number of transcription factors from S. cerevisiae have been identified that interact specifically with the 5′-CCCCT-3′ DNA motif via their C2H2 zinc finger domain(s). These transcription factors include Msn4p, Rgm1p, Rei1p, Rph1p, Msn2p, Gis1p, Com2p, and Usv1p (E value < 1). Comparison of these factors with proteins encoded by the Y. lipolytica genome (32) identified four C2H2 zinc finger domain-containing homologs (Table 2). The 5′-ACGCG-3′ motif interacts with the cell cycle regulator proteins Mbp1p, Swi6p, and Swi4p in S. cerevisiae (E value < 1) via an APSES DNA interaction domain (33). Comparison of these proteins with proteins encoded by the Y. lipolytica genome (32) identified two homologs (Table 2). We attribute the presence of fewer genes to the whole-genome duplication event in S. cerevisiae which generated many paralogs represented by a single gene in Y. lipolytica (34).

**TABLE 2:** Expression of *Y. lipolytica* genes predicted to regulate the *smooth* phenotype^*a*^

JGI protein ID	S. cerevisiae homolog(s)	Log_2_ fold change	*P* value
5′-CCCCT-3′ binding			
143137	*msn2*, *msn4*, *com2*	−2.63	3.46E−04
121652	*rei1*	0.90	4.68E−03
110816	*rph1*, *gis1*	0.61	4.76E−02
129649	*usv1*, *rgm1*	0.20	1.82E−01

5-ACGCG-3′ binding			
13938	*swi6*	0.84	2.98E−03
129847	*swi4*, *mbp1*	0.84	6.32E−03

a Fold change and *P* values represent the change in expression level between the *smooth-33* and wild-type strains during chemostat cultivation.

### Reverse genetics screen.

**FIG 5:**
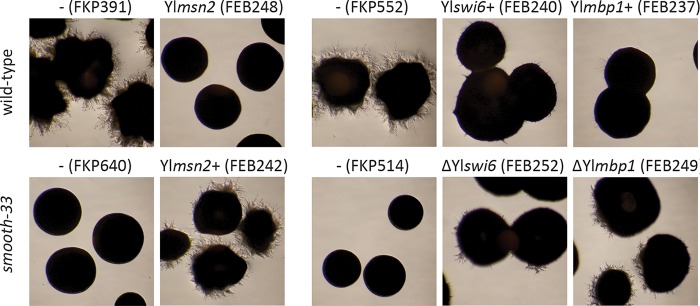
Ylmsn2p and the MBP complex regulate formation of hyphae. Ylmsn2p is predicted to interact with CCCCT promoter motifs, while the MBF complex (composed of Ylswi6p and Ylmbp1p) is predicted to interact with ACGCG motifs. Yl*msn2* was overexpressed in a *smooth-33* background and deleted in the parental hyphal background used for mutagenesis (FKP355). Conversely, Yl*swi6* and Yl*mbp1* were independently deleted in a *smooth-33* background and overexpressed in the parental background. Strains were cultured on YNB agar for 3 days at 28°C prior to examination of hyphae formation and imaging. Detailed genotypes are listed in Table 4.

We hypothesized that downregulation of genes with 5′-CCCCT-3′ promoter motifs in the *smooth-33* strain is controlled by a C2H2 zinc finger transcription factor. Of the four transcription factors predicted to bind this motif in Y. lipolytica, one (JGI protein ID 143137; Yl*msn2*) is very significantly downregulated (Table 2), which suggests that it may be an activator that is failing to regulate genes important for the yeast-to-hyphae transition in the *smooth-33* mutant. To test this, we overexpressed Yl*msn2* using a constitutive promoter in a *smooth-33* strain and deleted it in the wild-type parent used for the mutagenesis screen. We found that overexpression of Yl*msn2* restores hyphal growth in the *smooth-33* mutant, while deletion of Yl*msn2* results in loss of hyphal growth in wild-type Y. lipolytica, confirming its important role in regulation of this process and promotion of hyphal induction when expressed (Fig. 5).

We hypothesized that upregulation of genes with 5′-ACGCG-3′ promoter motifs in the *smooth-33* strain is controlled by Yl*swi6* (JGI protein ID 13938) and Yl*mbp1* (JGI protein ID 129847), which form a complex that regulates the G_1_/S phase transition in S. cerevisiae (35). Both of these genes are significantly upregulated in the *smooth-33* mutant (Table 2), suggesting promotion of the G_1_/S transition during yeast phase growth. We hypothesized that lower expression of these important cell cycle regulators in wild-type strains is associated with the transition to hyphal growth. To test this, we overexpressed Yl*swi6* or Yl*mbp1* using a constitutive promoter in the wild-type parent strain and deleted them in the *smooth-33* strain. We found that deletion of Yl*swi6* or Yl*mbp1* restores some hyphal growth in the *smooth-33* mutant, while overexpression of Yl*swi6* or Yl*mbp1* results in reduced hyphal growth in wild-type Y. lipolytica, confirming that these genes play a role in regulation of the yeast-to-hyphae transition process (Fig. 5).

### Isolation of mutants reverting to hyphal growth in the *smooth-33* background.

The success of our reverse genetic screen suggested that we may be able to identify additional factors regulating the yeast-to-hyphae transition via a forward genetic screen. Prototrophic Y. lipolytica *smooth-33* strain FKP514 was thus mutagenized with ethyl methanesulfonate (EMS) and plated on YNB agar plates to screen for colonies reverting to hyphal growth typical of wild-type Y. lipolytica on YNB. Approximately 500,000 colonies were screened, but no mutants were found that had reverted to a colony morphology typical of the wild type. However, 100 mutants were isolated that did not make completely smooth colonies. These mutants often appeared ruffled as colonies and upon microscopic observation appeared to have elongated cells and/or hyphae around their margins.

### Identification of mutations promoting the yeast-to-hyphae transition in the *smooth-33* background.

Twenty-eight of the hyphal mutants were sequenced using Illumina paired-end 150-bp sequencing and compared to the FKP355 reference genome to identify causative mutations. This initial search identified many genes with nonsynonymous mutations. Five genes were identified with nonsynonymous mutations in more than one mutant strain (JGI protein IDs 113409, 140296, 127631, 122144, and 109080), indicating that these genes are likely to be either the causative mutation or present at a hypermutable locus. The screen also identified four genes (JGI protein IDs 124736, 128138, 131882, and 129277) hit in only one mutant that are implicated in the yeast-hyphal transition in other species and present in a mutant with a low background mutation rate, indicating that they are likely to be the causative mutation (summarized in Table 3). Eight of the mutant strains had many nonsynonymous mutations, making prediction of a likely causative mutation difficult.

**TABLE 3:** High-confidence genes involved in yeast-to-hyphae transition^*a*^

JGI protein ID	S. cerevisiae BlastP^*b*^	No. of strains	Predicted mutations recovered
113409	*sln1* (*nik1*)	5	E342G, S441T, I536M, G584S, M598K
140296	*cts1*	4	K2*, W134*, G285E, G284V/E837D
127631	*ssk2*	3	G1190D, P555H, R526P
109080	*sln1* (*chk1*)	2	T1290M, E1415K
122144	*pbs2*	2	2 x G371R
124736	*hog1*	1	S335*
128138	*hym1*	1	L103P
131882	*lrg1*	1	G938C
129277	*mih1*	1	Y476*

a Genes with mutations in independent mutant strains as well as genes found in only one strain but with few or no other nonsynonymous mutations. Eight mutant strains contained many nonsynonymous mutations in unique gene hits and are not shown.

b Genes in parentheses represent the best BlastP hit from C. albicans.

**FIG 6:**
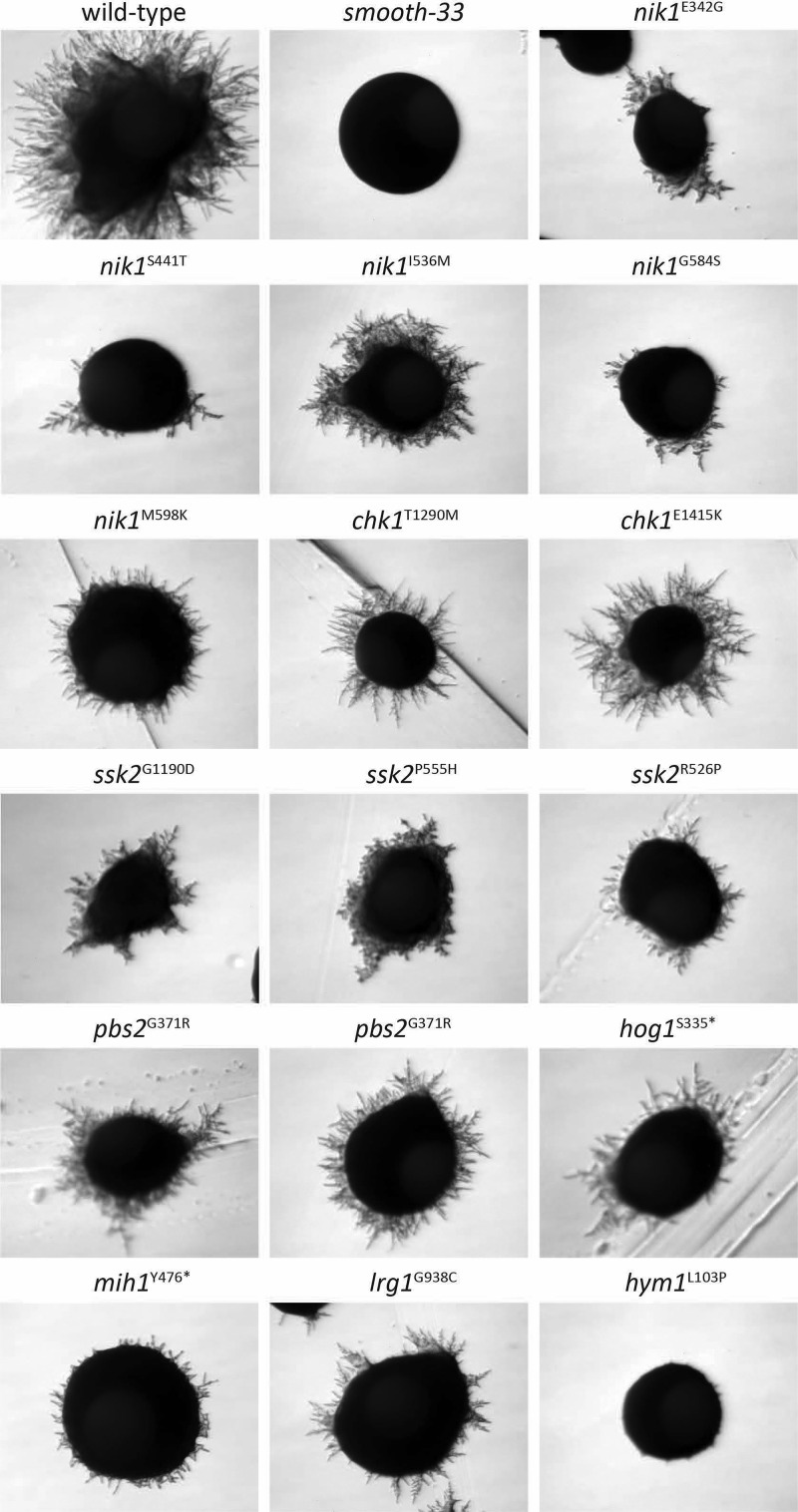
Mutants with a hyphal reversion phenotype in *smooth-33*. FKP514 (*smooth-33*) was mutagenized, and colonies exhibiting a transition to hyphal growth were isolated and sequenced. Mutant strains were plated on YNB agar, and isolated single colonies were imaged after 48 h at 28°C. Gene names shown are based on orthologs from S. cerevisiae and C. albicans. Mutations shown are the highest likelihood candidate identified after sequencing of each mutant.

**FIG 7:**
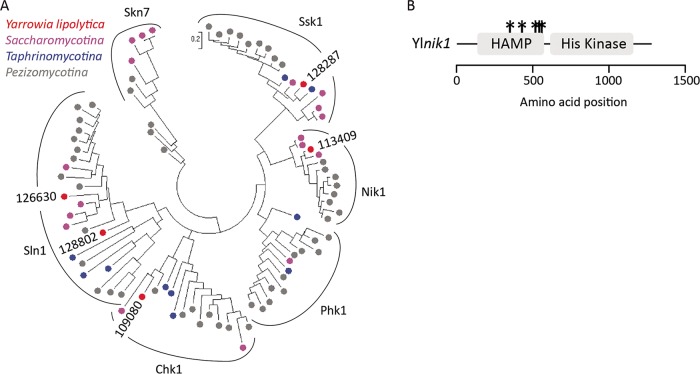
Histidine kinases in Y. lipolytica. (A) Phylogenetic reconstruction of selected histidine kinases from ascomycete fungi. Protein sequences from the histidine kinases of Y. lipolytica with similarity to Sln1p (FKP355 JGI protein ID 128287, 113409, 109080, 128802, and 126630) were used as bait to BlastP search the proteomes of Y. lipolytica, S. cerevisiae, C. albicans, Lipomyces starkeyi, Schizosaccharomyces pombe, Taphrina deformans, Ascobolus immerses, Monacrosporium haptotylum, Aspergillus nidulans, Stagnospora nodorum, Cladonia grayi, Botrytis cinerea, Neurospora crassa, and Xylona heveae. The BlastP hits were aligned using MUSCLE and analyzed by the maximum likelihood method with 200 bootstrap replicates to define the relationships between the Y. lipolytica genes and those from other species. (B) Protein domains from Ylnik1p were predicted by InterProScan (90). The kinase domain in Ylnik1p is predicted to be an S/T protein kinase. Note that all the mutations recovered occur in the HAMP domain. The sites of mutations are indicated by asterisks.

**FIG 8:**
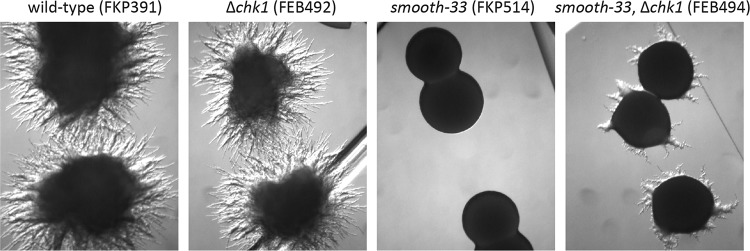
Yl*chk1* regulates formation of hyphae. Yl*chk1* was deleted in wild-type and *smooth-33* genetic backgrounds by replacement with *leu2*. Yl*chk1* is not required for the transition to hyphal growth morphology, but deletion results in limited reversion to hyphal morphology in *smooth-33*.

Five of the high-confidence gene hits appear to be homologous to genes in the high-osmolarity glycerol response (HOG) MAPK signaling pathway of S. cerevisiae. We recovered three independent alleles of the MAPK kinase kinase YL*ssk2*, two independent mutants with the same allele of the MAPK kinase Yl*pbs2*, and one mutant with a premature stop mutation in the MAPK Yl*hog1* (Fig. 6 and Table 3). In addition, we identified mutations in two genes with similarity to the *sln1* histidine phosphotransfer kinase, which regulates the HOG MAPK cascade in S. cerevisiae (36, 37). Further investigation into the structure of the mutated genes within the context of the histidine kinase gene family in Y. lipolytica revealed that proteins 113409 and 109080 (JGI protein IDs) are not orthologous to the *sln1*/*ssk1* two-component regulator (38) known to regulate the HOG MAPK cascade in S. cerevisiae. Rather, they represent proteins not found in S. cerevisiae that are orthologous to the *nik1* and *chk1* genes of C. albicans respectively (39, 40) (Fig. 7A). In C. albicans, both the histidine kinases *nik1* and *chk1*, as well as the *sln1* ortholog are involved in hyphal formation (41). Disruption of any of these genes impairs hyphal formation, while double disruption of *sln1* or *nik1* in combination with *chk1* partially restores hyphal formation (41). We disrupted Yl*chk1* in both the wild-type and *smooth-33* genetic background to assess its function in Y. lipolytica and to partially validate the results of the genetic screen. While Yl*chk1* is not required for hyphal formation, deletion in the *smooth-33* background partially restores hyphal formation consistent with the results obtained for the Yl*chk1* point mutants (Fig. 8).

The mutations isolated in Yl*nik1* are nonrandomly distributed (Fig. 7B). In Yl*nik1*, all five mutations occur within a series of HAMP domain repeats (42). These repeats are associated with fungicide sensing (43–46) and mutation of the HAMP domain in bacterial receptor histidine kinases is associated with constitutive activation (47–49). The very specific site of the five mutations present in Yl*nik1* from amino acids 342 to 598 (Table 3) and the lack of any putative nonfunctional mutations (e.g., premature stop codons or kinase functional domain mutations) suggests that Ylnik1p may be constitutively activated in these mutants and that the hyphal phenotype is caused by constitutive signaling rather than loss of function.

Three genes were recovered in single mutant strains known to be involved in morphogenesis in S. cerevisiae, including *hym1* (50), the GTPase-activating protein *lrg1* (51), and the tyrosine phosphatase *mih1* (52). Four independent mutations were found in the endochitinase *cts1* in strains with weaker colony morphology phenotypes typical of hyphal growth. Close observation revealed the morphology phenotype was due to a cell separation defect, as has been found in S. cerevisiae (53), rather than a switch to hyphal growth. These mutants were not considered further in this work.

## DISCUSSION

Development of yeast strains that do not switch between yeast and hyphal growth is critical for the utilization of fungi in reproducible bioprocesses. In this work, we isolated five spontaneous Y. lipolytica mutants that grow only in the yeast phase and do not form hyphae when cultivated on solid agar or in liquid medium in flasks or during bioreactor cultivation (Fig. 1). These mutants, which we named *smooth* mutants, were screened for rapid growth and nonreversion of the phenotype to identify strains useful for genetic engineering efforts toward the production of biofuels and chemicals with a Y. lipolytica host chassis. Genomic analysis of the *smooth* mutants revealed that all share mutation of a repetitive locus resulting in loss of short repetitive telomere-like DNA and rDNA repeats (Fig. 2 and 3). Transcriptome analysis of a selected mutant (*smooth-33*) revealed specific DNA motifs in the promoter regions of up- and downregulated genes (Fig. 4). These short DNA motifs implicated specific regulatory proteins important for maintenance of the yeast form which we confirmed by deletion and overexpression analysis (Fig. 5).

Our analysis identified the homolog of the stress response regulator Yl*msn2* as a primary regulator of the yeast-to-hyphae transition in *smooth* mutants. This gene, previously identified as *mhy1* in Y. lipolytica (23) is essential for the yeast-to-hyphae transition (Fig. 5) and activates gene expression in response to general stresses by binding to the stress response element 5′-CCCCT-3′ (23, 54). Loss of signaling through Yl*msn2* in the *smooth* strains and frequent hyphal growth in the wild-type strain suggests that our typical laboratory growth conditions (YNB medium at 28C) are stressful to Y. lipolytica. We observe more frequent initiation of hyphal growth on medium with a higher C/N ratio and less hyphae with a rich nitrogen source like peptone, which implicates nitrogen quantity and quality in this response (55). Msn2p is regulated by the TORC1-Sch9-Rim15 signaling pathway in Y. lipolytica (22), suggesting nutrient availability may be the inducer of hyphal growth in these experiments.

All five *smooth* strains exhibit what appear to be similar mutations in a poorly assembled, repetitive region of the genome represented by the end of scaffold 14 in the parent strain genome assembly (http://genome.jgi.doe.gov/Yarlip1/Yarlip1.home.html) (Fig. 2). Scaffold 14 ends in tandem 5′-TTAGTCAGGG-3′ repeats characteristic of Y. lipolytica telomeres (26); however, the mutation in some of the *smooth* strains, particularly the *smooth-43* strain, extends into the unique portion of scaffold 14 and initially alerted us to examine the repetitive DNA content of the wild-type and *smooth* strains. An unbiased search for short tandem repeats confirmed that the 5′-TTAGTCAGGG-3′ repeats are common and identified additional repetitive tandem DNA sequences present in the Y. lipolytica genome that are likely to constitute the telomere and subtelomere regions. Analysis of long PacBio reads found that many of these short repeats are bordered by rDNA repeats (28), and all repeat types are lost in similar quantity within each of the five *smooth* mutants (Fig. 3). Copy number variation in the rDNA repeats has been reported in filamentous fungi and yeasts and affects general physiological parameters, such as growth rate (56–59) and in C. albicans is associated with morphological mutants (60).

The complete mechanism governing loss of filamentous growth in the *smooth* mutants remains unclear. Our results indicate that expression changes in the *smooth* strains are governed primarily by reduced activation of genes with stress response elements by the transcription factor Msn2p (54, 61). Activation of the general stress response via Msn2p occurs through phosphorylation of the transcription factor by PKA and nuclear localization (62–64) and is dependent on cAMP signaling in response to a variety of nutritional and environmental stresses (65). We found that cell cycle progression genes are upregulated in the *smooth-33* mutant and that disruption of either component of the G_1_/S transition-promoting MBF complex (Mbp1p/Swi6p) (66, 67) conferred a sporadic low-level return to filamentous growth (Fig. 5). Together, these results suggest that the loss of repetitive telomeric and ribosomal DNA repeats is reducing signaling via the general stress response and promoting cell cycle progression.

We performed a forward genetic screen for reversion to hyphal growth in a prototrophic *smooth-33* strain to better understand the signaling occurring in response to the loss of repetitive telomeric and ribosomal DNA at the *smooth* locus (selected mutant phenotypes in Fig. 6). From this screen, 28 mutants were sequenced by high-throughput sequencing, and interestingly, we did not identify strains with mutations in Yl*mbp1* or Yl*swi6*. This suggests that the screen was not exhaustive for recovery of mutants with a sporadic reversion phenotype, as we sequenced the subset with the strongest hyphal phenotype maintained in all colonies after passaging and replating. Examination of the mutations in these strains implicates the histidine kinases Yl*nik1* and Yl*chk1* as well as the core components of the HOG MAPK cascade (Yl*ssk2*, Yl*pbs2*, and Yl*hog1*) in regulation of the yeast-to-hyphae transition in Y. lipolytica. In C. albicans, *nik1* and *chk1* are required for normal hyphal growth (41). Here we found that Yl*chk1* is not required for hyphal growth (Fig. 8). The mutations recovered in Yl*nik1* all occur only in the sensory HAMP domain (Fig. 7). Deletion of the HAMP domain in C. albicans *nik1* strain results in constitutive signaling as well as phosphorylation and activation of Hog1p (68). No mutations predicted to be nonfunctional were recovered in Yl*nik1*, suggesting that reversion to hyphal growth is due to constitutive or altered activation of this kinase.

Nik1p and Chk1p represent the common type III and type X histidine kinases that govern morphogenesis and enable pathogenicity in many fungi (69). Localization studies in Candida guilliermondii found that unlike the membrane-localized type VI histidine kinase, Sln1p, Nik1p, and Chk1p both localize to the cytosol and nucleus (70). While both Nik1p and Chk1p have been demonstrated to respond to general stresses by signaling to downstream targets, their method of sensing stresses has not been determined. Genetic studies have found that the nonkinase domains are required for sensing stresses, but further work is needed to determine how extracellular stresses alter the structure and activity of these cytoplasmic proteins (70). These histidine kinases, particularly *nik1*, are implicated in activation of the HOG pathway. However, it remains to be determined whether this affect is direct (e.g., through phosphorylation of HOG MAPK components) or a result of stress caused by their constitutive signaling.

In summary, we examined Y. lipolytica mutants that do not transition to hyphal morphology under conditions relevant to industrial production of biofuels and commodity chemicals. We identified mutations in the repetitive DNA of these strains that reduce signaling through the general stress response pathway via an unknown mechanism. Reversion to hyphal growth is possible in these mutants via signaling or lack thereof by Yl*msn2*, the HOG MAPK cascade components (Yl*ssk2*, Yl*pbs2*, and Yl*hog1*), and the histidine kinases encoded by Yl*nik1* and Yl*chk1*. This work builds upon our understanding of the dimorphic transition in Y. lipolytica and confirms that the pathways regulating this morphological switch are conserved with other ascomycete yeasts. How the loss of repetitive DNA reduces the *msn2*-mediated stress response remains an enigma. Eleven of the mutants recovered in the reversion to hyphal growth screen warrant further analysis and may shed light on the connection between the loss of repetitive DNA and reduction of the stress response.

## MATERIALS AND METHODS

### Yeast cultivation and forward genetic screens for nonhyphal mutants.

All Y. lipolytica strains used in this study (Table 4) were maintained in YNB (1.7 g/liter yeast nitrogen base without amino acids and ammonium sulfate but with 20 g/liter glucose and 5 g/liter ammonium sulfate) or YPD (10 g/liter peptone, 10 g/liter yeast extract, 20 g/liter glucose) liquid medium at 28°C and 200 rpm unless otherwise noted. Auxotrophs were supplemented with 0.1 g/liter leucine when appropriate. Frozen stocks were maintained at −80°C in 15% glycerol. To isolate *smooth* mutants, Y. lipolytica strain FKP355 was passaged daily in YPD for 2 weeks to allow accumulation of mutations and plated at a density of 10,000 cells per plate on YNB agar plates. Plates were incubated 72 h at 28°C to allow development of colonies. Large colonies without hyphae were streaked onto fresh YNB plates to obtain pure mutant strains. Purified mutant strains were inoculated onto YPD, YNB, and YNB150 (1.7 g/liter yeast nitrogen base without amino acids and ammonium sulfate but with 25 g/liter glucose and 0.367 g/liter ammonium sulfate) agar plates to confirm the phenotype. To isolate *smooth* mutants reverting to hyphal growth, Y. lipolytica strain FKP514 was mutagenized with ethyl methanesulfonate (EMS) (71) and plated at a density of 10,000 cells per plate on YNB agar plates. Plates were incubated 72 h at 28°C to allow development of colonies. Colonies exhibiting ruffled morphologies characteristic of the transition to hyphal growth were streaked onto fresh YNB plates to obtain pure mutant strains. Chemostat cultivation was performed with a dilution rate of 0.05 per hour at 30°C in a 1.2-liter bioreactor (DASGIP, Jülich, Germany) with a working volume of 750 ml at pH 3.5, controlled with 2 M KOH. Dissolved oxygen was kept above 30% with a stirrer rate of 600 rpm and an airflow rate of 1 v.v.m. The growth medium contained 25 g/liter glucose, 0.5 g/liter (NH_4_)_2_SO_4_, 5.96 g/liter K_2_SO_4_, 3 g/liter KH_2_PO_4_, 0.5 g/liter MgSO_4_·7H_2_O, vitamins and trace metal solutions (72) and 125 μl antifoam 204 (Sigma-Aldrich, St. Louis, MO, USA). Samples for transcriptomic analysis were taken when the chemostats reached steady state, defined as stable CO_2_ and O_2_ outflow and optical density, which was achieved after circa 120 h.

**TABLE 4:** *Y. lipolytica* strains used in this study

Strain	Genotype	Reference
FKP355	*matA leu2-270 xpr2-332 axp-2 ku70*::*hph*^+^	55
FKP391	*matA leu2-270*::*leu2*^+^ *xpr2-332 axp-2 ku70*::*hph*^+^	55
FKP500	*matA leu2-270 xpr2-332 axp-2 ku70*::*hph*^+^ *smooth-17*	This work
FKP501	*matA leu2-270 xpr2-332 axp-2 ku70*::*hph*^+^ *smooth-18*	This work
FKP502	*matA leu2-270 xpr2-332 axp-2 ku70*::*hph*^+^ *smooth-19*	This work
FKP503	*matA leu2-270 xpr2-332 axp-2 ku70*::*hph*^+^ *smooth-33*	This work
FKP504	*matA leu2-270 xpr2-332 axp-2 ku70*::*hph*^+^ *smooth-43*	This work
FKP514	*matA leu2-270*::*leu2*^+^ *xpr2-332 axp-2 ku70*::*hph*^+^ *smooth-33*	This work
FEB248	*matA leu2-270 xpr2-332 axp-2 ku70*::*hph*^+^ *msn2*::*leu2*^+^	This work
FKP552	*matA leu2-270 xpr2-332 axp-2 ku70*::*hph*^+^ *exp1p*-:*leu2*^+^	This work
FEB237	*matA leu2-270 xpr2-332 axp-2 ku70*::*hph*^+^ *exp1p*-*mbp1*:*leu2*^+^	This work
FEB240	*matA leu2-270 xpr2-332 axp-2 ku70*::*hph*^+^ *exp1p*-*swi6*:*leu2*^+^	This work
FKP640	*matA leu2-270 xpr2-332 axp-2 ku70*::*hph*^+^ *smooth-33 exp1p*-:*leu2*^+^	This work
FEB242	*matA leu2-270 xpr2-332 axp-2 ku70*::*hph*^+^ *smooth-33 exp1p*-*msn2*:*leu2*^+^	This work
FEB249	*matA leu2-270 xpr2-332 axp-2 ku70*::*hph*^+^ *smooth-33 mbp1*::*leu2*^+^	This work
FEB252	*matA leu2-270 xpr2-332 axp-2 ku70*::*hph*^+^ *smooth-33 swi6*::*leu2*^+^	This work
FKP672	*matA leu2-270*::*leu2*^+^ *xpr2-332 axp-2 ku70*::*hph*^+^ smooth-33 mih1^Y476*^	This work
FKP673	*matA leu2-270*::*leu2*^+^ *xpr2-332 axp-2 ku70*::*hph*^+^ *smooth-33 lrg1^G938C^*	This work
FKP675	*matA leu2-270*::*leu2*^+^ *xpr2-332 axp-2 ku70*::*hph*^+^ *smooth-33 nik1^E342G^*	This work
FKP677	*matA leu2-270*::*leu2*^+^ *xpr2-332 axp-2 ku70*::*hph*^+^ *smooth-33 nik1^S441T^*	This work
FKP681	*matA leu2-270*::*leu2*^+^ *xpr2-332 axp-2 ku70*::*hph*^+^ *smooth-33 nik1^I536M^*	This work
FKP682	*matA leu2-270*::*leu2*^+^ *xpr2-332 axp-2 ku70*::*hph*^+^ *smooth-33 nik1^G584S^*	This work
FKP683	*matA leu2-270*::*leu2*^+^ *xpr2-332 axp-2 ku70*::*hph*^+^ *smooth-33 nik1^M598K^*	This work
FKP684	*matA leu2-270*::*leu2*^+^ *xpr2-332 axp-2 ku70*::*hph*^+^ *smooth-33 pbs2^G371R^*	This work
FKP686	*matA leu2-270*::*leu2*^+^ *xpr2-332 axp-2 ku70*::*hph*^+^ *smooth-33 ssk2^G1190D^*	This work
FKP687	*matA leu2-270*::*leu2*^+^ *xpr2-332 axp-2 ku70*::*hph*^+^ smooth-33 hog1^R335*^	This work
FKP689	*matA leu2-270*::*leu2*^+^ *xpr2-332 axp-2 ku70*::*hph*^+^ *smooth-33 chk1^T1290M^*	This work
FKP690	*matA leu2-270*::*leu2*^+^ *xpr2-332 axp-2 ku70*::*hph*^+^ *smooth-33 ssk2^P555H^*	This work
FKP691	*matA leu2-270*::*leu2*^+^ *xpr2-332 axp-2 ku70*::*hph*^+^ *smooth-33 ssk2^R526P^*	This work
FKP694	*matA leu2-270*::*leu2*^+^ *xpr2-332 axp-2 ku70*::*hph*^+^ *smooth-33 chk1^E1415K^*	This work
FKP695	*matA leu2-270*::*leu2*^+^ *xpr2-332 axp-2 ku70*::*hph*^+^ *smooth-33 pbs^G371R^*	This work
FKP730	*matA leu2-270*::*leu2*^+^ *xpr2-332 axp-2 ku70*::*hph*^+^ *smooth-33 hym1^L103P^*	This work
FEB492	*matA leu2-270 xpr2-332 axp-2 ku70*::*hph*^+^ *chk1*::*leu2*^+^	This work
FEB494	*matA leu2-270 xpr2-332 axp-2 ku70*::*hph*^+^ *smooth-33 chk1*::*leu2*^+^	This work

### Reference genome sequencing and assembly.

Genomic DNA and RNA were isolated from Y. lipolytica strain FKP355 (55) using a yeast genomic DNA purification kit (AMRESCO, Solon, OH) and TRIzol reagent (Invitrogen, Carlsbad, CA), respectively. One microgram of DNA was sheared to 10 kb using the g-TUBE (Covaris). The sheared DNA was treated with DNA damage repair mix followed by end repair and ligation of SMRT adapters using the PacBio SMRTbell Template Prep kit (PacBio). The SMRTbell templates were then purified using exonuclease treatments and size selected using AMPure PB beads. Sequencing primer was then annealed to the SMRTbell templates, and Version P6 sequencing polymerase was bound to them. The prepared SMRTbell template libraries were then sequenced on a Pacific Biosciences RSII sequencer using Version C4 chemistry and 4-h sequencing movie run times. Filtered subread data were assembled together with Falcon version 0.4.2 (https://github.com/PacificBiosciences/FALCON) to generate an initial assembly. Mitochondria were then assembled separately using the corrected preads with Celera version 8.3 and subsequently polished with Quiver. It was then used to remove mitochodrial data from the preads. A secondary Falcon assembly was generated using the filtered preads with Falcon version 0.4.2 and polished with Quiver version smrtanalysis_2.3.0.140936.p5 (https://github.com/PacificBiosciences/GenomicConsensus). The final genome assembly was annotated using the JGI Annotation Pipeline (73).

Stranded cDNA libraries were generated using the Illumina Truseq Stranded RNA LT kit. mRNA was purified from 1 μg of total RNA using magnetic beads containing poly(T) oligonucleotides. mRNA was fragmented and reverse transcribed using random hexamers and SSII (Invitrogen) followed by second strand synthesis. The fragmented cDNA was treated with end pair, A-tailing, adapter ligation, and eight cycles of PCR. The prepared Illumina libraries were quantified using KAPA Biosystem’s next-generation sequencing library qPCR kit and run on a Roche LightCycler 480 real-time PCR instrument. The quantified libraries were then multiplexed with other libraries, and the pool of libraries was then prepared for sequencing on the Illumina HiSeq 2500 sequencing platform utilizing a TruSeq paired-end cluster kit, v4, and Illumina’s cBot instrument to generate a clustered flow cell for sequencing. Sequencing of the flow cell was performed on the Illumina HiSeq2500 sequencer using a TruSeq SBS sequencing kit, v4, following a 2 × 100 indexed run recipe.

Transcriptome raw fastq file reads were evaluated for artifact sequence using BBDuk (https://sourceforge.net/projects/bbmap/), raw reads by kmer matching (kmer = 25), allowing 1 mismatch and detected artifact was trimmed from the 3′ ends of the reads. RNA spike-in reads, PhiX reads, and reads containing any Ns were removed. Quality trimming was performed using the phred trimming method set at Q6. Finally, following trimming, reads under the length threshold were removed (minimum length 25 bases or 1/3 of the original read length, whichever is longer). Filtered fastq files were used as input for *de novo* assembly of RNA contigs. Reads were assembled into consensus sequences using Trinity (ver. 2.1.1) (74) with the –normalize_reads (In-silico normalization routine) and –jaccard_clip (Minimizing fusion transcripts derived from gene dense genomes) options. The assembled transcriptome was used for genome annotation and made available through the JGI fungal genome portal MycoCosm (http://genome.jgi.doe.gov/Yarlip1/Yarlip1.home.html).

### Genome resequencing and identification of mutations.

Genomic DNA was prepared from wild-type and mutant strains using a yeast genomic DNA purification kit (AMRESCO, Solon, OH) followed by 150-bp paired-end sequencing on an Illumina MiSeq instrument or 100-bp paired-end sequencing on an Illumina HiSeq instrument (San Diego, CA). The paired-end reads were aligned to the Y. lipolytica FKP355 reference genome sequence available at the website http://genome.jgi.doe.gov/Yarlip1/Yarlip1.home.html using BWA (75) or Bowtie2 (76) and visualized with the Integrated Genomics Viewer (77). Mutations were identified and annotated with Samtools (78), Pindel (79), BreakDancer (80), CNVnator (81), SnpEff (82), and custom Perl scripts.

### Overexpression plasmid construction.

Overexpression plasmids were constructed using pYL15 as a vector (55). Coding sequences from YL*msn2*, Yl*mbp1*, and Yl*swi6* were PCR amplified using primer pairs OEB491/492, OEB497/498, and OEB503/504, respectively, from Y. lipolytica FKP355 genomic DNA using Q5 DNA polymerase (New England Biolabs, Ipswich, MA) (Table 5). Plasmid pYL15 was digested with SmaI and Fast AP (Fermentas, Waltham, MA) to dephosphorylate plasmid ends. The PCR products were purified using a GeneJET purification kit (Thermo Fisher Scientific, Waltham, MA) and assembled using the NEBuilder HiFi assembly kit (New England Biolabs, Ipswich, MA) according to the manufacturer’s instructions to produce autonomously replicating overexpression plasmids for *msn2*, *mbp1*, and *swi6*.

**TABLE 5:** Primers used in this study

Primer	Sequence (5→3′)
OKP443	ACCCGTTGCTATCTCCACAC
OKP444	GTGCAGTCGCCAGCTTAAA
OEB491	ATATCTACAGCGGTACCCCCATGGACCTCGAATTGGAAAT
OEB492	CCGCCTCCGCCGATATCCCCCTAGTCCCGAGGATGCGTA
OEB497	ATATCTACAGCGGTACCCCCATGTCCATCTACAAAGCAAC
OEB498	CCGCCTCCGCCGATATCCCCCTATCTCTCTCCCTCAAGCA
OEB503	ATATCTACAGCGGTACCCCCATGCCCGACGTGAAACACGA
OEB504	CCGCCTCCGCCGATATCCCCTCATGCCTGCTGAGGAGGCT
OEB544	CTGATCGTACCTTGATGTCGACCCGTTGCTATCTCCACAC
OEB545	CGTACAGTTCGAGGATCGTAGTGCAGTCGCCAGCTTTAAA
OEB487	GGTTTTGAGTCTTGGGAGTGG
OEB548	CGACATCAAGGTACGATCAGATGGGCCAAAGTTAGTGGTG
OEB549	TACGATCCTCGAACTGTACGCCTTCTAGTCTCCGCTCCAT
OEB490	CCACAGCTGCTCTTATGACG
OEB493	GTAGTTTCGGTTGCCTCGTC
OEB550	CGACATCAAGGTACGATCAGTCGAGTTACCCTATGTGCTG
OEB551	TACGATCCTCGAACTGTACGGGGTCGGTCTAGGACGATGT
OEB496	GACACAAAGCTCATCGGTGG
OEB499	TGCAATCTCCTCCCAGATTT
OEB552	CGACATCAAGGTACGATCAGTGTCGTGAACGTCTTTGAGC
OEB553	TACGATCCTCGAACTGTACGCTCACGGTATGGGCTGTTCT
OEB502	TCTCCGAGGCCATCATTTAG
OEB846	TTGATCCTGATGGTCGTGAA
OEB847	CGACATCAAGGTACGATCAGATCAGCGGAGATGTTTCGTC
OEB848	TACGATCCTCGAACTGTACGGAATAAACCGTCAGCCCAGA
OEB849	GGCGACACAGTCAGAGCATA
OEB4	CGGAGATGATATCGCCAAAC
OEB575	GAGCTGCCATTGAGAAGGAG

### Yeast strain construction.

Transformations were performed by the lithium acetate method (83), and transformants were selected on YNB agar. PCR products were amplified using Q5 DNA polymerase (New England Biolabs, Ipswich, MA) and custom primers (Table 5). DNA fragments were purified using a GeneJET purification kit (Thermo Fisher Scientific, Waltham, MA). *leu2*-*270* was complemented in FKP503 (*smooth-33*) by transformation with full-length *leu2* after PCR amplification using primer pair OKP443/444 to construct strain FKP514. Integration at the *leu2-270* locus was confirmed by PCR. Yl*msn2*, Yl*mbp1*, Yl*swi6*, and Yl*chk1* were replaced with a *leu2*^+^ nutritional marker. Briefly, 1-kb regions flanking each gene were amplified from FKP355 genomic DNA using Q5 DNA polymerase and primers designed with overhangs homologous to the *leu2* gene (amplified with primers OEB544/545) from Y. lipolytica genomic DNA (primer pairs OEB487/548, OEB549/490, OEB493/550, OEB551/496, OEB499/552, OEB553/502, OEB846/847, and OEB848/849). The fragments were purified using a GeneJET purification kit (Thermo Fisher Scientific, Waltham, MA) and assembled into full-length deletion cassettes with *leu2* using NEBuilder HiFi assembly kit or as split marker deletion cassettes with internal *leu2* primers OEB4 and OEB575. Deletion cassettes were transformed into strain FKP355 or FKP503 as appropriate. Replacement of genes with *leu2*^+^ was confirmed by PCR. Deletion and overexpression strains were characterized on YNB agar at 28°C.

### Transcriptome analysis.

Samples for transcriptome analysis were collected from steady-state chemostats, frozen in liquid nitrogen, and stored at −80°C. Total RNA was extracted with TRIzol (Invitrogen, Carlsbad, CA, USA) following the manufacturer’s instructions with additional mechanical disruption of the cells using a FastPrep homogenizer (MP Biomedicals, Santa Ana, CA, USA) and 1-mm silica beads. Further RNA preparation and RNA sequencing were performed by SciLifeLab in Uppsala, Sweden, using their IonTorrent platform. Raw RNA-seq reads were aligned to the Y. lipolytica genome using Bowtie (76), and counts were obtained with HTSeq (84) and transformed using voom (85). The top 1,000 genes with the greatest positive and negative fold change values from the FKP514 versus FKP391 transcriptome comparison were analyzed for enrichment of Gene Ontology terms using FunRich (86). The 500-bp promoter region of the top 1,000 genes with the greatest positive and negative fold change values from the FKP514 versus FKP391 transcriptome comparison were analyzed for enrichment of specific sequence motifs using DREME (87). Identified motifs were compared to the Jaspar core fungal motifs database (31) using Tomtom (88) to identify candidate regulators.

### Microscopy.

For confocal microscopy, live cells were collected and immediately visualized using a Zeiss LSM710 confocal laser-scanning microscope (Carl Zeiss MicroImaging GmbH, Munchen, Germany) with a Plan-Apochromate 100×/1.4 oil objective. All images were processed using ImageJ (89). For colony morphology, cells were imaged on a VWR Stereo Zoom Trinocular microscope fitted with a Canon EOS 6D DSLR camera, and images were processed with Adobe Photoshop.

### Data availability.

Sequence data from the whole-genome shotgun project for Y. lipolytica FKP355 have been deposited at DDBJ/ENA/GenBank under accession number PKSB00000000. The version of sequence data described in this paper has accession number PKSB01000000. Sequence data for the Y. lipolytica *smooth* strains (FKP355 and FKP500 to FKP504) have been deposited at NCBI SRA under accession number PRJNA499126. Sequence data for the Y. lipolytica hyphal reversion strains (FKP514 to FKP730) have been deposited at NCBI SRA under accession numbers SRP145806, SRP145808, SRP145807, SRP145813, SRP145810, SRP145811, SRP145814, SRP145809, SRP145815, SRP145817, SRP145820, SRP145818, SRP145816, SRP145821, SRP145825, SRP145822, SRP145824, SRP145826, SRP145835, SRP145830, SRP145834, SRP145828, SRP145836, SRP145832, SRP145831, SRP145829, SRP145833, SRP145837, and SRP145838. Transcriptome data have been deposited at ArrayExpress under accession number E-MTAB-7400.
